# Linking optical and molecular signatures of dissolved organic matter in the Mediterranean Sea

**DOI:** 10.1038/s41598-017-03735-4

**Published:** 2017-06-13

**Authors:** Alba María Martínez-Pérez, Mar Nieto-Cid, Helena Osterholz, Teresa S. Catalá, Isabel Reche, Thorsten Dittmar, Xosé Antón Álvarez-Salgado

**Affiliations:** 1 Consejo Superior de Investigaciones Científicas - Instituto de Investigacións Mariñas (CSIC-IIM), Vigo, Spain; 2 Research Group for Marine Geochemistry, Institute for Chemistry and Biology of the Marine Environment (ICBM), Carl von Ossietzky University, Oldenburg, Germany; 3 Departamento de Ecología and Instituto del Agua, Universidad de Granada, Granada, Spain

## Abstract

Dissolved organic matter (DOM) plays a key role in global biogeochemical cycles and experiences changes in molecular composition as it undergoes processing. In the semi-closed basins of the oligotrophic Mediterranean Sea, these gradual molecular modifications can be observed in close proximity. In order to extend the spatial resolution of information on DOM molecular composition available from ultrahigh resolution mass spectrometry in this area, we relate this data to optical (fluorescence and absorption spectroscopy) measurements. Covariance between molecular formulae signal intensities and carbon-specific fluorescence intensities was examined by means of Spearman’s rank correlations. Fifty two per cent of the assigned molecular formulae were associated with at least one optical parameter, accounting for 70% of the total mass spectrum signal intensity. Furthermore, we obtained significant multiple linear regressions between optical and intensity-weighted molecular indices. The resulting regression equations were used to estimate molecular parameters such as the double bond equivalent, degradation state and occurrence of unsaturated aliphatic compounds from optical measurements. The statistical linkages between DOM molecular and optical properties illustrate that the simple, rapid and cost-efficient optical spectroscopic measurements provide valuable proxy information on the molecular composition of open ocean marine DOM.

## Introduction

Marine dissolved organic matter (DOM) comprises a complex mixture of molecules essentially uncharacterized and present at very low concentrations^1, 2^. It is well established that DOM plays a key role in marine biogeochemical cycles: it constitutes the primary source of nutrients and energy for heterotrophic growth^3^, regulates the UV and visible light absorption^4^, undergoes photochemical processing^5^, acts as a trace metal ligand^6^ and presents antioxidant activity for minimizing the negative effects of free radicals in aquatic organisms^7^ among other services to the marine ecosystems. DOM is mainly produced in the ocean epipelagic layer (0–150 m depth) as a result of phytoplankton photosynthesis and subsequent food web interactions^8^. Most of this recently produced DOM is biologically labile and thus has a short lifetime^9, 10^. However, a small fraction of that DOM escapes rapid mineralization because it is originally resistant or it is transformed into resistant materials through abiotic processes or during the microbial processing of bioavailable DOM^11^. This DOM which escapes rapid remineralization accumulates in the surface layer for eventual export to the dark ocean (>150 m depth) by convective overturning and vertical mixing^12, 13^.

A small fraction of DOM absorbs light in the UV and visible range of the spectrum; it is called colored DOM (CDOM) and it is present in the ocean ubiquitously^4^. Furthermore, a fraction of CDOM emits fluorescence; it is called fluorescent DOM (FDOM) and it is widely used to trace the reactivity, composition^14–17^, sources and chemical structure of DOM^18–22^. Fourier Transform Ion Cyclotron Resonance Mass Spectrometry (FT-ICR-MS) is a widely used technique nowadays to distinguish thousands of molecular formulae constituting the DOM pool. Although this technique does not unambiguously reveal the structure of DOM compounds, it offers new possibilities for the characterization of individual formulae or classes of molecules. The linkage between optical and chemical properties of DOM in natural waters is a subject of rising interest among aquatic biogeochemists^23^. Recent studies have combined FT-ICR-MS and optical measurements to better understand the composition of DOM in natural waters^24, 25^ but few of them have directly related optical and molecular signatures^26–31^. These studies, mainly focused on freshwater and coastal systems, have reported significant relationships between optical indices and molecular formulae. However, DOM of terrestrial and marine origin is characterized by contrasting molecular size and composition, as well as absorption and fluorescence spectral properties^4, 32^. Therefore, the results obtained in previous studies conducted in freshwater and coastal systems should not be directly generalized to open ocean waters.

In this study, we link the fluorescence and absorption of DOM with the molecular signatures obtained from the FT-ICR-MS data of 29 samples from the open epi–, meso– and bathypelagic waters of the Mediterranean Sea. Our main objective is to determine the covariance between optical and molecular signatures in this low-CDOM ocean system and examine similarities and differences with the relationships found in high-CDOM freshwater and coastal systems. Furthermore, we aim to go a step ahead by establishing semi-quantitative relationships between an assortment of calculated optical and molecular indices. Our final goal is the use of fluorescence and absorption spectroscopy measurements as a proxy for the indices obtained from molecular data, which are more time demanding and costly to obtain. Note that these relationships do not imply a direct causation but the existence of a common cause or causes of variation between optical and molecular indices.

## Material and Methods

### Sampling strategy

**Figure 1:**
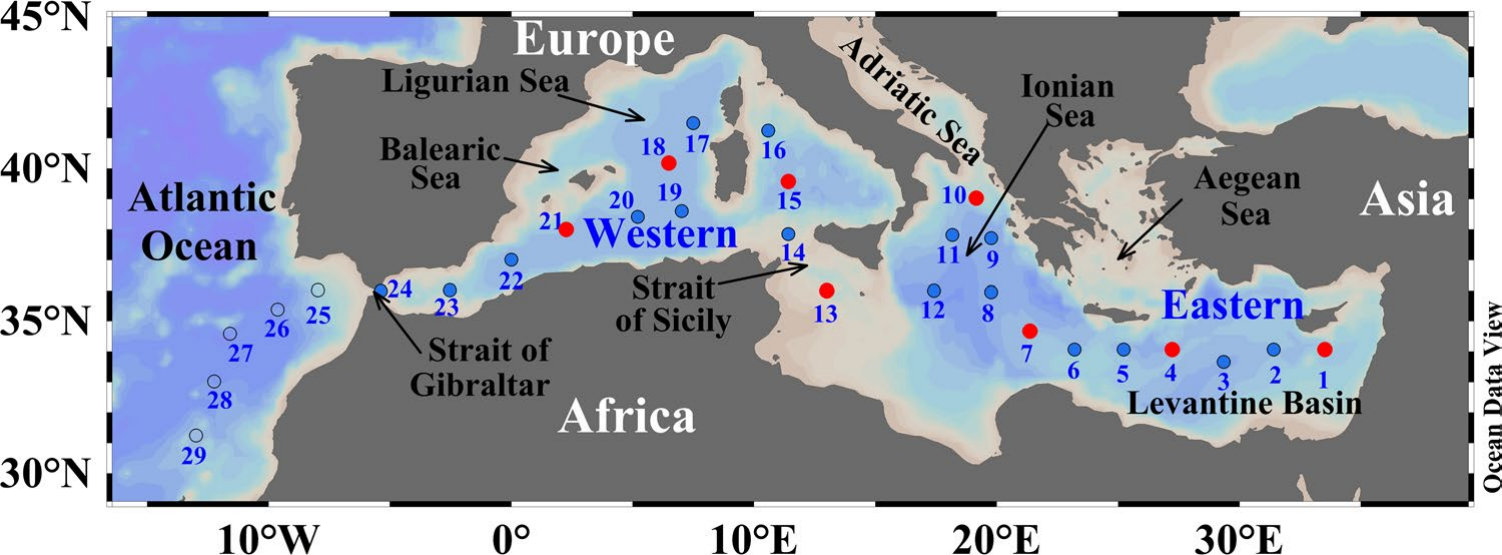
Study area and sampling stations. The circles depict all the cruise stations. Red circles represent the stations where samples were taken for the molecular characterization of DOM. Red and blue circles represent the stations where samples were collected for DOM fluorescence and absorption characterization. Figure created using Ocean Data View (version 4.7.8, R. Schlitzer, 2016. http://odv.awi.de).

During the trans-Mediterranean cruise HOTMIX (see cruise track in Fig. 1) aboard R/V Sarmiento de Gamboa (Heraklion, Crete, 27 April 2014 – Las Palmas, Canary Islands, 29 May 2014), water samples were collected using a SBE 38 rosette sampler, equipped with 24 (12 L) Niskin bottles. Conductivity, temperature and depth probes (CTD SBE 911 plus) as well as oxygen (SBE-43 oxygen sensor) and fluorescence (SeaPoint fluorometer) sensors were attached to the rosette. Distributions of salinity, potential temperature, dissolved oxygen and chlorophyll *a* are shown in Figure S1. We used these profiles to sample the deep chlorophyll maximum, the salinity maximum, the oxygen minimum layer and the deep waters. For the determination of dissolved organic carbon (DOC), optical properties, and molecular formulae, seawater samples were collected in 5-litres acid-cleaned polycarbonate carboys. Prior to filtration (within 5 hours), samples were stored in the dark at 13 °C and then filtered through precombusted (450 °C, 4 h) Whatman GF/F filters. Two-liter aliquots of the filtrate were collected in acid-cleaned PTFE bottles for solid phase extraction of DOM (SPE-DOM).

### Dissolved organic carbon and optical measurements

Aliquots of 10 mL of the filtrate were collected for DOC quantification in precombusted (450 °C, 12 h) glass ampoules. These samples were acidified with H_3_PO_4_ (85%, p.a., Merck) to pH < 2 and the ampoules were flame sealed and stored in the dark at 4 °C until analysis in the base laboratory. DOC concentration was determined with a Shimadzu TOC-V organic carbon analyzer by high temperature catalytic oxidation (HTCO). Potassium hydrogen phthalate (99.95–100.05%, p.a., Merck) was used to calibrate the system daily. The precision of the equipment was ±1 μmol L^−1^. The accuracy was checked daily with the DOC reference materials provided by D. A. Hansell (University of Miami, USA).

Absorbance of CDOM was measured on board using a double beam Perkin Elmer lambda 850 spectrophotometer equipped with 10 cm path length quartz cuvettes for sample and reference (ultrapure water, Milli-Q, Millipore Advantage A10). Spectral scans were collected from 250 nm to 750 nm at 1 nm intervals and constant room temperature. The absorbance was converted into Naeperian absorption coefficient (m^−1^)^33^. The carbon-specific absorption coefficient at 254 nm (*a*254*; L m^−1^ mg^−1^ C) was calculated.

FDOM was determined on board with a Perkin Elmer LS55 luminescence spectrometer. Slit widths were 10 nm for both excitation and emission wavelengths. Measurements were performed at room temperature in a 1 cm quartz fluorescence cell and Milli-Q was used as a reference blank. The spectrofluorometer was tested daily: intensity of the Raman peak was tested with a sealed Milli-Q water cell (Perkin Elmer) while p-terphenyl and tetraphenylbutadiene methacrylate blocks (Starna) were used to check the instrument signal intensity at the excitation-emission wavelengths characteristic of the aromatic amino acids and humic-like substances, respectively. Excitation (Ex)-emission (Em) matrices (EEMs) were generated by combining 22 fluorescence emission spectra from 300 to 560 nm at excitation wavelengths ranging from 240 to 450 nm at 10 nm intervals. Rayleigh scatter bands were removed mathematically from the EEMs. Parallel factor analysis (PARAFAC) was used to decompose the fluorescence signal of the EEMs into the underlying individual fluorescent components^34^. Data analyses were performed using the DOMFluor 1_7 Toolbox8 and conducted in Matlab R2014B (MathWorks, USA). A four-component model with the most common fluorescent peaks of aquatic environments was obtained (Figure S2), two of them of humic-like nature, compatible with peaks A + C (at Ex/Em < 270–350/470 nm) and M (at Ex/Em 330/402 nm) and two of amino acid-like nature, attributed to tryptophan and tyrosine at Ex/Em 280/374 nm and 270/306 nm, respectively. Model validation was performed by split-half diagnostics and random initialization^35, 36^.

Given that the PARAFAC components (Figure S2) match the classical fluorescence peak intensities at selected Ex/Em wavelength pairs previously established^37^, we decided to use the latter to allow direct comparison with previous studies. The selected Ex/Em wavelengths were 250/435 nm (peak A) due to general humic substances; 340/440 nm (peak C) due to humic substances of terrestrial origin; 320/410 nm (peak M) due to humic substances of marine origin; and 280/350 nm (peak T) and 270/304 nm (peak B) due to protein-like substances. The fluorescence of each peak was determined by subtracting the Milli-Q blank peak height from the sample average peak height. All the samples were normalized using a solution of quinine sulphate dihydrate (≥99.0%, purum for fluorescence, Fluka) and tryptophan (≥99.0%, Fluka) standards in H_2_SO_4_ 0.05 M (95–97%, p.a., Merck) allowing to express fluorescence in Normalized Fluorescence Intensity Units (NFIU)^38^. The fluorescence of each peak was then calculated as the mean of 4 individual measurements, which presented a coefficient of variation of <6 ± 2% (n = 30) for all the peaks. Carbon-specific fluorescence of humic-like (peaks A*, C* and M*) and protein-like (peak T*) substances were calculated dividing the fluorescence intensity by the DOC concentration (in NFIU L mg^−1^ C). Humic-/protein-like and terrestrial/marine fluorescence ratios (A/T, C/T, M/T and C/M) were also calculated. In addition, we calculated fluorescence-based indices such as the fluorescence (FI)^39^, freshness (FrI)^29^, biological (BIX)^40^ and humification (HIX)^41^ indices (see Table 1 for wavelength ranges).

**Table 1:** Positive correlations between the total assigned molecular formulae as well as the different type of molecular groups and the optical parameters; carbon-specific fluorescence intensity in NFIU L mg^−1^ C of general humic-like substances (peak A*), terrestrial humic-like substances (peak C*), marine humic-like substances (peak M*), protein-like substances (peak T*), fluorescence ratios (peak A/T, C/T and M/T ratios), carbon-specific absorption coefficient at 254 nm (*a*254*; L m^−1^ mg^−1^ C), fluorescence index (FI) calculated as the ratio of emission at 470 and 520 nm at excitation wavelength 370 nm, freshness index (FrI) calculated as the ratio between 380 and the maximum intensity between 420 and 435 nm at 310 emission wavelength, biological index (BIX) determined as the ratio of emission at 380 and 430 nm at 310 nm of excitation wavelength and the humification index (HIX) calculated as the ratio between the integrated emission spectra between 435 and 480 and 300–345 nm at 260 nm of excitation wavelength. NC = no correlation. MW = intensity-weighted average molecular weight, Nr = number. Numbers in parentheses correspond to the signal intensity percentage from the spectra.

	All	NC	Peak A*	Peak C*	Peak M*	Peak T*	A/T	C/T	M/T	C/M	*a*254*	FI	FrI	BIX	HIX
Nr total formulae	3689 (100)	1995 (35)	408 (27)	511 (30)	432 (25)	704 (19)	396 (27)	447 (29)	429 (28)	72 (2)	718 (20)	441 (12)	825 (22)	843 (23)	298 (22)
MW (Da)	375	366	426	430	433	315	420	421	422	350	317	289	313	314	398
Nr formulae with N	1456 (100)	756 (52)	135 (10)	156 (10)	133 (8)	328 (24)	140 (11)	161 (12)	153 (11)	35 (4)	324 (24)	222 (16)	389 (28)	390 (29)	112 (12)
Black Carbon (Nr)	8 (100)	3 (23)	0 (0)	0 (0)	0 (0)	3 (45)	0 (0)	0 (0)	0 (0)	0 (0)	2 (32)	3 (47)	4 (62)	0 (0)	3 (0)
Polyphenols (Nr)	433 (100)	220 (47)	32 (16)	36 (16)	30 (11)	93 (13)	35 (17)	39 (18)	37 (18)	7 (2)	93 (14)	74 (12)	124 (19)	121 (19)	46 (23)
Highly unsaturated (Nr)	2594 (100)	1460 (35)	293 (29)	279 (32)	317 (27)	448 (17)	288 (29)	316 (32)	305 (31)	56 (2)	454 (18)	271 (11)	509 (20)	523 (21)	193 (22)
Unsaturated aliphatic (Nr)	507 (100)	238 (30)	73 (29)	83 (30)	75 (28)	122 (23)	70 (27)	76 (28)	73 (27)	5 (4)	133 (24)	74 (15)	144 (26)	152 (28)	51 (23)
Saturated fatty acids (Nr)	28 (100)	10 (11)	0 (0)	1 (1)	0 (0)	13 (62)	0 (0)	0 (0)	0 (0)	0 (0)	13 (62)	7 (38)	16 (66)	17 (88)	0 (0)
Sugars (Nr)	30 (100)	20 (32)	3 (35)	3 (35)	3 (35)	2 (8)	4 (23)	6 (52)	6 (52)	0 (0)	2 (8)	2 (8)	3 (11)	3 (11)	2 (18)
Peptides (Nr)	89 (100)	44 (33)	7 (15)	9 (17)	7 (15)	23 (30)	7 (15)	10 (17)	8 (16)	4 (4)	21 (29)	10 (9)	25 (42)	24 (42)	6 (6)

### Solid phase extraction and FT-ICR-MS analysis

For SPE-DOM isolation, filtered seawater samples (2 L) were acidified to pH 2 and the DOM was extracted on board using PPL cartridges (Agilent)^42^. After extraction, cartridges were rinsed with acidified ultrapure water to remove remaining salts and frozen at −20 °C. Once in the base lab, the cartridges were dried by flushing with high purity N_2_ and eluted with 6 mL of methanol (HPLC-grade, Sigma-Aldrich). SPE-DOM methanol extracts were diluted with ultrapure water and methanol (MS grade) to yield a DOC concentration of 15 mg C L^−1^ and a methanol-to-water ratio of 1:1 (v/v) for analysis by ultrahigh-resolution mass spectrometry using a Solarix FT-ICR-MS (Bruker Daltonik GmbH) connected to a 15 Tesla superconducting magnet (Bruker Biospin). Molecular formulae were assigned to the detected masses^43^. The signal intensity of each identified molecular formula was normalized to the sum of all molecular formula intensities with signal-to-noise ratio (S/N) higher than 5 in each sample, so we can interpret the FT-ICR-MS data semi-quantitatively^44^. The degree of unsaturation of a compound was assessed based on its molecular formula and was expressed as the double bond equivalent (DBE)^45, 46^ and the degradation index (Ideg) was calculated^47^. The identified molecular formulae were assigned to compound groups based on established molar ratios, modified aromaticity index, DBE and heteroatom contents as described elsewhere^43^. This assignment into molecular compound groups is not an unambiguous method for molecular structure determination, but it provides a useful overview of likely structures behind a given set of molecular formulae. The averages per sample, weighted by relative intensity of the detected masses, of molecular weight (MW), DBE and abundance of compound groups were also calculated. The reproducibility and the detection limit of the FT-ICR-MS method was assessed in previous works^48, 49^ by performing multiple measurements of the same reference sample. This sample, collected at a depth of 670 m at the Natural Energy Laboratory of Hawaii Authority (NELHA) in Kona, Hawaii^50^, was also used as an internal reference sample to assess and correct for instrument variability over time. In our work, the detection limit was established at a fixed signal-to-noise ratio (S/N) higher than 4. In addition, we tested the reproducibility of the peak detection using NELHA sample replicates (n = 13) and found a coefficient of variation of 13 ± 6%.

### Statistical analyses and graphical tools

**Figure 2:**
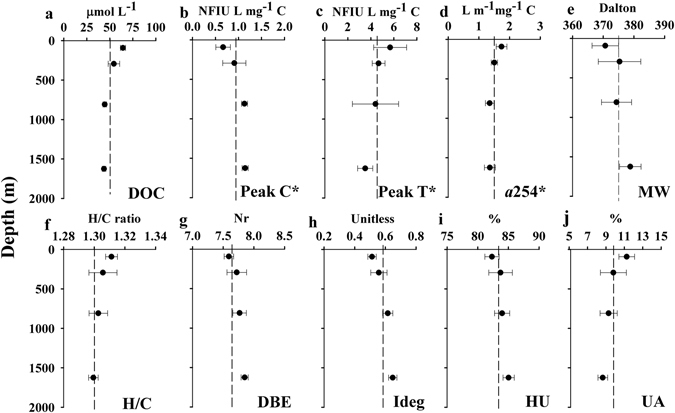
Vertical profiles of DOC, optical and molecular parameters averaged by water layer (**a**) DOC, (**b**) carbon-specific fluorescence intensity of humic-like substances (peak C*), (**c**) carbon-specific fluorescence intensity of protein-like substances (peak T*), (**d**) carbon-specific absorption coefficient at 254 nm (*a*254*), (**e**) average molecular weight (MW), (**f**) H/C ratio, (**g**) double bond equivalent (DBE), (**h**) degradation index (Ideg), (**i**) relative abundance of the highly unsaturated (HU) and (**j**) relative abundance of unsaturated aliphatic (UA) compounds. Error bars are standard deviation and the dashed lines represent the mean value for each variable. Figure created using the software Sigma plot (version 11.0).

**Figure 3:**
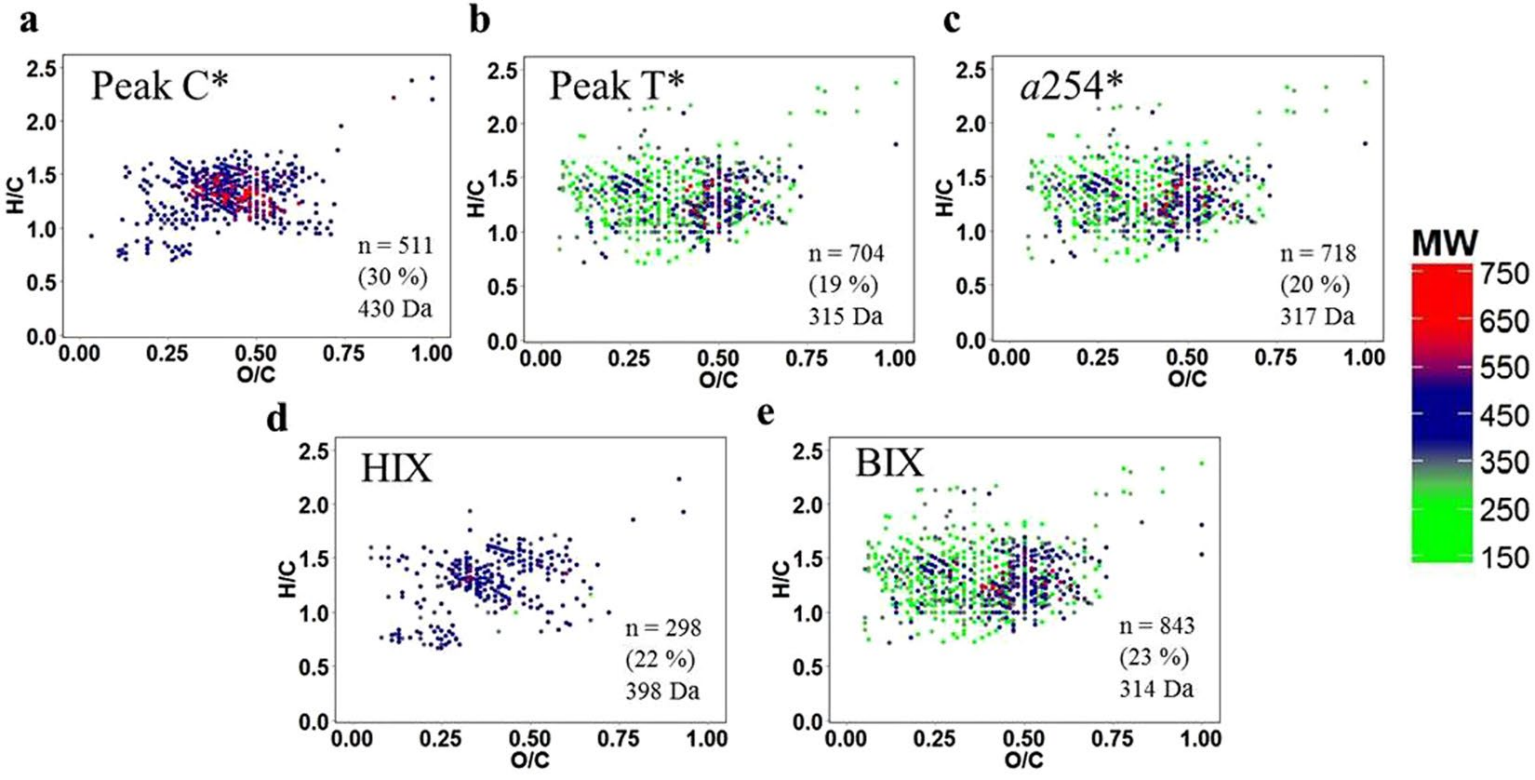
Van Krevelen diagrams. Molecular formulae positively correlated with (**a**) peak C*, (**b**) peak T*, (**c**) a254*, (**d**) HIX and (**e**) BIX. Color scale represents the molecular weight. In the right-bottom corner of each panel is summarized the number of molecular formulae correlating, the percentage of peak intensity and the average molecular weight. Figure created using R software (version 3.1.1, 2014-07-10, http://cran.r-project.org/).

**Figure 4:**
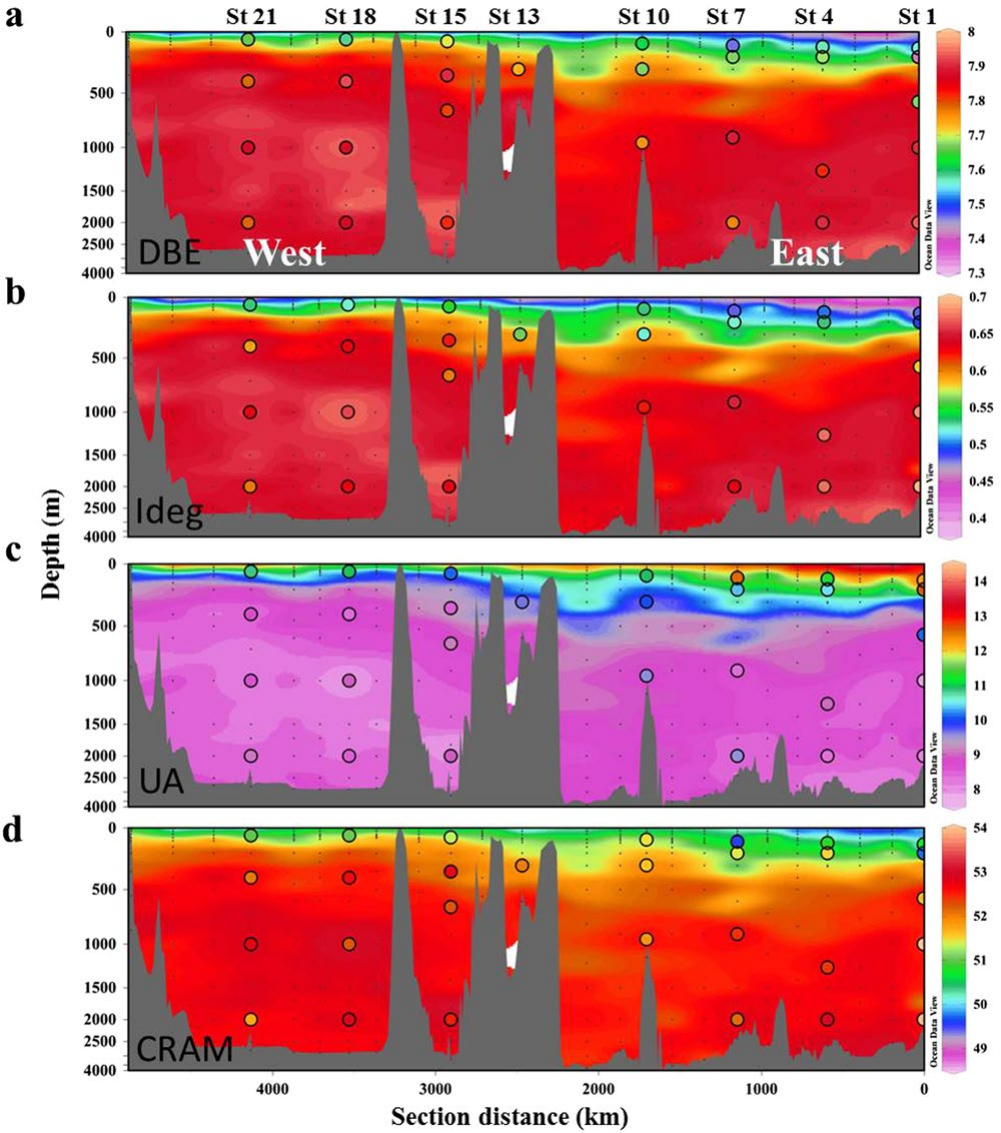
Distributions of the estimated molecular parameters. (**a**) double bond equivalent (DBE) in number, (**b**) degradation index (Ideg) unitless, (**c**) unsaturated aliphatic compounds (UA) in % and (**d**) carboxyl-rich alicyclic molecules (CRAM) in % for the whole Mediterranean Sea determined from fluorescence measurements. Colored dots represent molecular parameters calculated by FT-ICR-MS analysis. Figure created using Ocean Data View (version 4.7.8, R. Schlitzer, 2016. http://odv.awi.de).

**Figure 5:**
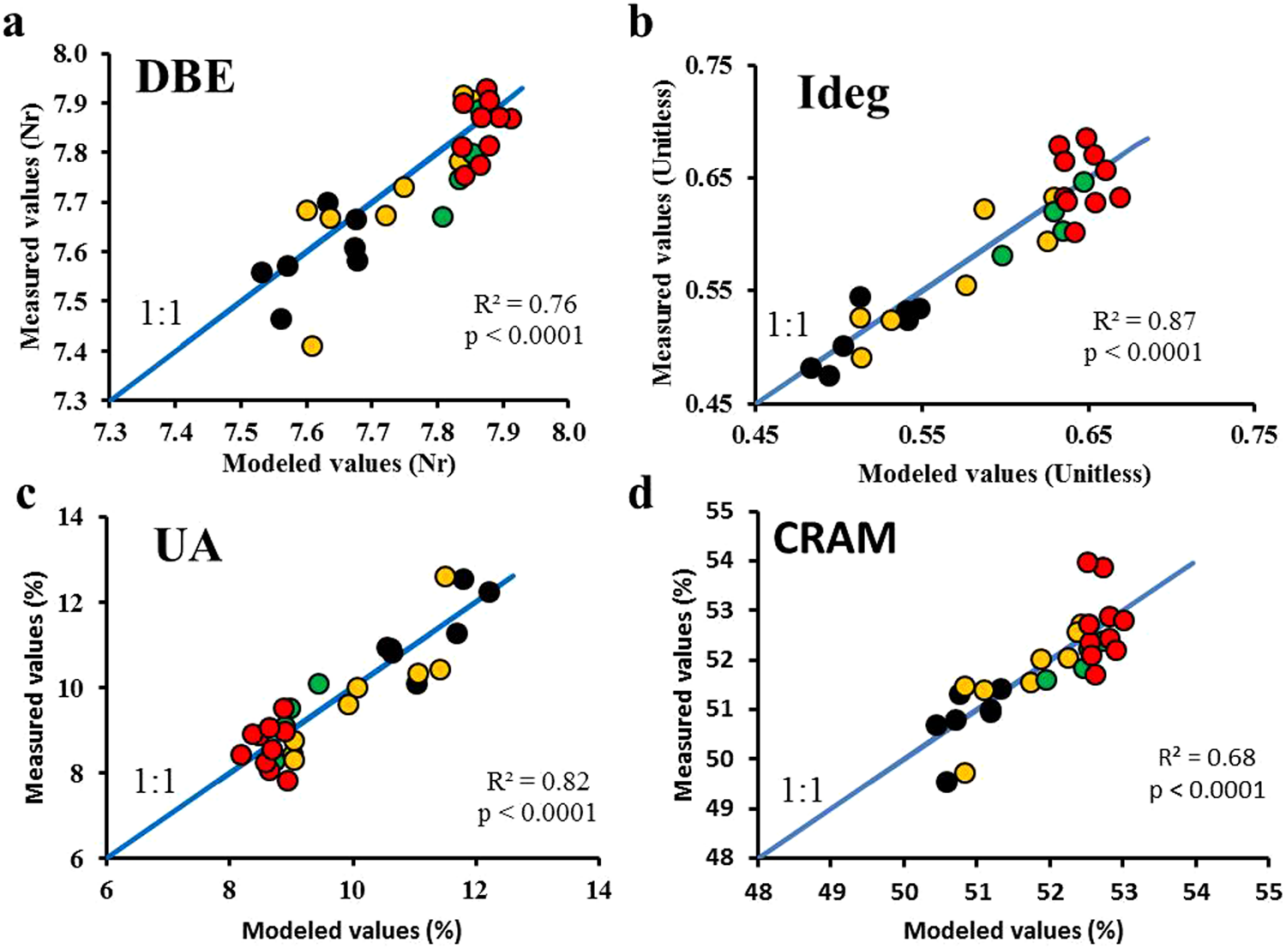
Comparison of modeled and measured molecular DOM properties. The modeled values were calculated using the multiple linear regression models and the measured values were obtained from the FT-ICR-MS analysis for (**a**) double bond equivalent (DBE), (**b**) degradation index (Ideg), (**c**) unsaturated aliphatic compounds and (**d**) carboxyl-rich alicyclic molecules (CRAM). Colored dots represent samples collected at different layers, deep chlorophyll maximum (black), Levantine intermediate water (yellow), oxygen minimum layer (green) and deep waters (red). Note that there is a random deviation from the 1:1 line (blue line). For the CRAM regression (plot **d**) red dots with higher measured values correspond with samples collected at station 1 and the black and yellow dots, with lower measured values, correspond with samples collected at station 7 and 1, respectively.

Carbon-specific fluorescence peak intensities, fluorescence ratios, *a*254*, FI, FrI, HIX and BIX were correlated with all assigned molecular formulae applying Spearman’s rank correlations. Each individual optical parameter was correlated with each individual molecule. Spearman’s rank correlation coefficients (r) greater than 0.43 were considered significant at the 99% confidence limit (Student’s t-test for a set of 29 samples). Therefore, all molecular formulae correlating with r > 0.43 with a given optical index were assigned to that optical index. A similar approach was applied in previous studies^26, 27, 29^. In addition, multiple linear regression models between molecular parameters and optical indices were calculated using Pearson’s regressions. To determine if our sample size allows to detect significant differences we performed a power analysis for n = 29. We found that the power was higher than 0.72 for all variables. We therefore assume that the sample size n = 29 is appropriate to detect significant differences. To validate our models we performed a cross validation exercise. We used 80% of the samples to obtain the models and then validated them with the remaining 20% of the samples. The subsets to obtain and validate the models were chosen randomly and repeated 1000 times. Then, we calculated the error of the estimation for the 1000 runs and obtained mean errors. All the statistical analyses were performed in R^51^ (version 3.1.1, 2014-07-10, http://cran.r-project.org/). Figure 1 was created using the Ocean Data View (ODV) software^52^, Fig. 2 was created using Sigma plot (version 11.0), Fig. 3 was produced using the R (version 3.1.1, 2014-07-10), Fig. 4 was created using the ODV software and Fig. 5 was produced using Microsoft Excel 2010.

## Results and Discussion

### Carbon stock and optical characterization of DOM in the Mediterranean Sea

DOC concentrations of the collected samples showed the characteristic exponential decay profile with depth found in the open ocean. Considering the sampling depths, maximum concentrations higher than 60 μmol C L^−1^ were observed at the deep chlorophyll maximum (DCM) and minimum concentrations of about 42 μmol C L^−1^ were found in the deep waters (Fig. 2a). These results are in agreement with previous studies in the Mediterranean Sea^53, 54^. The vertical distribution of the C-specific fluorescence of humic-like substances (peak C*) increased with depth although leveling off from about 750 m to the bottom (Fig. 2b). The increase of peak C* with depth was likely caused by the production of humic-like materials during microbial respiration processes in meso- and bathypelagic layers^16, 55–57^ and its accumulation due to the refractory nature (centennial turnover times) of this material^17, 58^. The peak C* minimum at the DCM can also be attributed to the vulnerability of humic-like substances to photobleaching in the upper mixed layer^15, 59^. Conversely, the vertical profile of the carbon-specific fluorescence of protein-like substances (peak T*) was opposite to peak C* (Fig. 2c). The maximum value for peak T* at the sampling depths was found at the DCM and attributed to the accumulation of semi-labile materials, such as amino acids from phytoplankton exudation, cell autolysis and microzooplankton grazing^60, 61^. In addition, those substances are much less vulnerable to photodegradation than humic-like substances^62^, which leads to their accumulation in this layer. The observed decrease of peak T* with depth likely resulted from the bio-labile nature of these compounds, as it was suggested in a previous study^63^, who found a strong relationship between protein-like fluorescence and biodegradable DOC. In our study, the peak T* profile was also similar to the DOC profile, showing a similar pattern to other ocean areas^14^. This finding indicates that peak T* at the DCM accumulates at a higher rate than the bulk DOM and it would be consumed faster in the deep ocean. The carbon-specific absorption coefficient at 254 nm (*a*254*), used as an aromaticity index^18^, showed a decreasing profile with depth, although it remained constant from about 750 m to the bottom (Fig. 2d), similar to the DOC and peak T* profiles (Fig. 2a,c).

The FI reveals information about DOM origin, terrestrial versus microbial^64^, and ranged between 1.8 and 2.2. This result indicates that FDOM in the Mediterranean Sea presented a predominantly microbial origin^21^. FrI and BIX, both indicators of recent biological activity^29, 40^, ranged between 0.8 and 1.0. HIX, a proxy to the DOM humification degree, ranged between 0.9 and 7.6. The values of these indices are indicative of weak humic character and important recent autochthonous sources of DOM^40^. FI, FrI and BIX showed a vertical profile similar to *a*254*, DOC and peak T*. However HIX was, as expected, more similar to the profile of peak C*.

### Molecular level DOM characterization in the Med Sea

A total of 6057 resolved molecular masses of singly charged compounds were detected in the FT-ICR-MS spectra covering a mass range of 154–817 Da. We assigned 3689 molecular formulae in the mass range of 157–736 Da, not considering ^13^C isotopologues. The vertical profile of the molecular parameters calculated from the mass spectra (Fig. 2e–j) showed that DOM compounds were more unsaturated and of higher molecular weight with depth. As it has been suggested, the MW increases from recent to aged material^47^. In addition, lower H/C and higher O/C ratios have been observed as indicators of reworked organic matter^47, 65^. The downwards increasing profiles of DBE, degradation index and the abundance of highly unsaturated compounds together with the decrease in unsaturated aliphatic compounds and H/C ratio indicated an increase of the unsaturation degree with depth. These results are in agreement with previous studies^47, 66^. Highly unsaturated compounds increased with depth as they are likely produced during the remineralization processes in the meso- and bathypelagic layers. On the contrary, maximum proportions of unsaturated aliphatic compounds were found at the DCM as they comprise a major fraction of phytoplankton exudates^66^. These compounds are then presumably consumed throughout the water column due to their bioavailability. The most abundant group of molecules, in terms of number of formulae, was represented by the highly unsaturated compounds (70.4 ± 0.6%), followed by unsaturated aliphatics (13.7 ± 0.7%), polyphenols (11.8 ± 0.2%), peptides (2.5 ± 0.1%), saturated fatty acids with heteroatoms (0.78 ± 0.05%) and sugars with heteroatoms (0.6 ± 0.1%). Carboxyl-rich alicyclic molecules (CRAM)^67^ accounted for 21.9 ± 0.6% of the total number of molecular formulae. Note that 94% of the molecular formulae assigned to CRAM were also classified as highly unsaturated compounds. All assigned molecular formulae were represented in a van Krevelen diagram (Figure S3) grouped by compound classes.

### Linking optical and molecular properties of SPE-DOM

We explored the covariability of each one of the 3689 molecular formulae identified from the FT-ICR-MS analysis with the C-specific fluorescence intensities, fluorescence indices and absorption coefficients described in the Material and Methods section by means of Spearman’s rank correlations (99% confidence limit). Our statistical analysis indicated that 52% of the 3689 assigned molecular formulae were correlated, either positively or negatively, with one or more optical parameter. These molecular formulae accounted for 70% of the mass spectrum peak intensities. Considering only the positive correlations (*r* > 0.43), 46% of the molecular formulae (65% of the spectra signal intensity) correlated with one or more optical parameter (Table 1). These percentages were slightly higher than those found in Canadian boreal rivers^27^, but lower than in the Florida Everglades^29^. Note that the number of molecular formulae not correlating (NC) with any optical parameter was different for the positive and negative correlations (Tables 1 and S1, respectively). This is due to the fact that a molecular formulae that correlated positively with one or more optical parameters but did not correlate negatively with any optical parameter will count in the no correlation group in Table S1 (negative correlations) but not in Table 1 (positive correlations). The majority of the formulae correlated significantly with more than one optical parameter (Table S2). These results indicate that fluorescence and absorption measurements covary with a substantial fraction of the SPE-DOM and not only with the minor fraction of fluorescent molecular formulae. Note that the fractions of DOM captured by both techniques are different, and both techniques have specific analytical windows. For optical measurements only the colored/fluorescent pools of DOM are considered. Furthermore, FDOM can be affected by quenching processes, which depend on the sample matrix^68^. FT-ICR-MS analysis, on the other hand, provides information on the solid-phase extractable and ionizable fraction. In this regard, SPE-DOM comprises a wide range of the most apolar DOM molecules to highly polar molecules, but not the smallest ionic molecules (i.e. short chain organic acids and free amino acids) and colloidal aggregates^69^. ESI is a soft ionization technique that preferentially ionizes polar functional groups that renders DOM molecules their water solubility^70^.

A statistical linkage between molecular formulae and optical properties does not necessarily imply that compounds with a given molecular formula contain fluorophores or chromophores, but it indicates similar biogeochemical behavior in an aquatic system^28^.

The number of positive correlations (*r* > 0.43) between the molecular formulae and humic-like compounds and fluorescence ratios (peak A/T, C/T and M/T) were fewer compared to the negative correlations (*r* < −0.43), however they accounted for higher relative spectrum intensities (Tables 1 and S1). On the contrary, the number of molecules positively correlated with peak T*, *a*254*, FrI and BIX was much higher than the number of negative correlations but they accounted for relatively lower spectrum intensities. Plotting the significant correlations into Van Krevelen diagrams (Fig. 3), where the color scale represents the molecular weight of each formula, we can distinguish the molecular families associated with each optical parameter. For the range of materials isolated with our SPE methodology, which excludes free amino acids and colloidal aggregates (see above), we observed that peak C* correlated positively with higher average MW formulae and peak T* correlated with lower average MW formulae. *a*254* and BIX correlated positively with formulae similarly to peak T* whereas HIX was related to peak C* (Fig. 3). Van Krevelen diagrams representing the negative correlations for the same variables (Figure S4) showed the opposite trend, peak C* and HIX correlated with lower average MW molecules and peak T*, *a*254* and BIX were related to higher average MW molecules.

We also studied the correlations between different groups of molecules and the optical indices (Table 1). We observed that about 59% of the spectrum intensity assigned to polyphenols correlated with one or more optical parameter. These compounds were negatively correlated with peak M*, fluorescence ratios A/T, C/T and M/T and BIX and positively with peak T*, *a*254* and HIX. These findings are in agreement with the fact that some polyphenols fluoresce in an area relatively close to the protein-like substances region^58, 71^. Furthermore, it was suggested that hydrocarbons are detected in the peak T* region^27^.

About 70% of the mass spectrum intensity of highly unsaturated compounds, the most abundant group in our samples (Figure S5a), correlated both positively and negatively with all the optical parameters (Tables 1 and S1). The unsaturated aliphatic compounds were the group that accounted the most to the correlations with the optical properties (about 75% of their mass spectrum intensity). Almost the same percentage of these compounds correlated positively and negatively with peaks A*, C*, M* and the fluorescence ratios A/T, C/T and M/T (Tables 1 and S1). However, the positive correlations were greater compared with the negative for peak T*, likely due to the labile nature of these compounds as it was suggested in previous studies^15, 22, 63^, and for some of the fluorescence indices (FI, FrI and BIX) which are proxies for recent biologic activity.

The saturated fatty acids correlated only negatively with peak A*, C* and M*, the ratios A/T, C/T and M/T and HIX and positively with peak T*, *a*254*, FI, FrI and BIX. Similarly, peptides correlated negatively with peak A*, C*, M*, the ratios A/T, C/T, M/T and HIX, but only positively with peak T* and *a*254*. These results are in agreement with the fact that saturated fatty acids are presumably bioavailable molecules^72^.

The distribution of all the molecules positively correlated with peak C* and peak T* are graphically shown in Figure S5b and c. Compared with the mean compound group distribution of SPE-DOM in the Mediterranean Sea (Figure S5a), molecules correlating with peak C* were enriched in highly unsaturated compounds and impoverished in polyphenols. Conversely, molecules correlating with peak T* were enriched in unsaturated aliphatic molecules but impoverished in highly unsaturated molecules. In addition, peak T* showed an enrichment in polyphenols compared to peak C*.

### Inferring DOM molecular indices from optical properties

Once we have verified the existence of significant correlations between DOM molecular formulae and optical properties, we propose using multiple linear regressions to estimate molecular indices from optical indices. First, a correlation matrix including salinity (S), potential temperature (θ), peak C*, peak T* and a254* was obtained (data not shown) to check for covariance between these variables. On basis of this analysis, Peak C* and peak T* along with θ were chosen for performing the multiple regressions. These regression models were fitted using the 29 samples for which we have peak C* and peak T* measurements together with FT-ICR-MS molecular parameters. The cross validation exercise of the multiple regression of each molecular index with peak C*, peak T* and θ (see Materials and Methods) yielded estimated mean errors of 1, 5, 7 and 1% for DBE, Ideg, UA and CRAM, respectively. Therefore, the models were able to estimate molecular parameters from optical indices with a relatively low error. Then, we calculated the mean and standard deviation of the regression coefficients obtained after the 1000 times that each regression model was run to perform the cross validation exercise. These mean regression coefficients (Table 2) were not significantly different from the regression coefficients obtained with the individual multiple linear regressions of the molecular indices with peak C*, peak T* and θ for the 29 samples. The regression coefficients showed that peak C* was positively correlated with DBE, Ideg and CRAM, but negatively with unsaturated aliphatic compounds. Opposite results were obtained with peak T*. Regarding the normalized regression coefficients (beta), they were higher for peak C* than for peak T* for all the regressions (Table 2). The relative weight of peak T* increases when modeling Ideg and CRAM.

**Table 2:** Mean values (standard deviation) of the coefficients of the multiple linear regressions (Pearson) for n = 1000 between the intensity-weighted average molecular parameters from FT-ICR-MS analysis of the SPE-DOM (DBE = double bond equivalent^46^, Ideg = degradation index^47^, unsaturated aliphatic compounds (UA) and CRAM = carboxyl rich alicyclic molecules)^67^ and potential temperature and the optical properties of the DOM (carbon-specific fluorescence intensity of the humic-like compounds (peak C*) and the protein-like compounds (peak T*) in NFIU L mg^−1^ C. *beta* = Normalized regression coefficients, SEE = standard error of estimate in %.

	Peak C*	Peak T*	θ	Intercept	SEE	R^2^	p- value
DBE	0.32 (0.12); *beta* = 0.55	−0.02 (0.01); *beta* = −0.22	−0.02 (0.02); *beta* = −0.24	7.89 (0.47)	1.0	0.73	<0.0001
Ideg	0.13 (0.03); *beta* = 0.50	−0.016 (0.003); *beta* = −0.30	−0.0015 (0.006); *beta* = −0.31	0.75 (0.11)	4.6	0.85	<0.0001
UA	−2.5 (0.9); *beta* = −0.44	0.23 (0.07); *beta* = 0.22	0.39 (0.18); *beta* = 0.39	5.5 (3.4)	6.7	0.80	<0.0001
CRAM	1.8 (0.7); *beta* = 0.43	−0.24 (0.05); *beta* = −0.30	−0.21 (0.15); *beta* = −0.27	54.2 (2.7)	1.1	0.65	<0.0001

The mean regression coefficients in Table 2 were then used to estimate the molecular parameters of the 400 samples collected during the cruise for the determination of peak C* and peak T* (Fig. 4). Since the latter were performed with a JY-Horiba Spex Fluoromax-4 spectrofluorometer, the measurements of the 29 samples determined with both spectrofluorometers were used to intercalibrate both instruments (R^2^ = 0.88, p < 0.0001 for peak C and R^2^ = 0.77, p < 0.0001 for peak T). Using θ, peak C* and peak T* we were then able to estimate molecular indices (DBE, Ideg, unsaturated aliphatic compounds and CRAM) and build up detailed maps of their distribution for the whole Mediterranean Sea. The model goodness is confirmed by the low mean error of the estimates from the cross validation exercise, by Fig. 4 where colored dots represent measured values for the molecular parameters, and by Fig. 5 where we compared the measured and modeled values using linear regressions. These results indicate that the multiple regression models work well for the Mediterranean Sea (R^2^ > 0.65, p < 0.0001) and reliable detailed spatial molecular information from FDOM measurements can be gained. Regarding these distributions (Fig. 4), the upper layer in the eastern basin showed SPE-DOM with lower DBE, degradation state and CRAM than the western basin but presented more abundance of unsaturated aliphatic compounds. Note that for the CRAM distribution (Fig. 4d) the mismatches in the samples collected at station 1 (200, 1000 and 2000 m) and station 7 (110 m) were due to the fact that in the scatter plot (Fig. 5d) the points exhibited a stronger divergence from the linear regression line. Although differences between measured and modeled values of % CRAM in these samples were apparent, the other molecular parameters (i.e. Ideg, AImod, and DBE) did not show such differences. To check if these samples could lead to a different result in the model, we discarded these samples as outliers to obtain a new model and new estimated values. Comparing the two model results (Figure S6), the new model presented slightly lower values in some deep regions but the patterns were not different than in the former model. For this reason we decided to keep these samples in the model.

Overall, in this work we have shown that optical and molecular properties correlated significantly in the Mediterranean Sea. In detail, we have observed relationships between different SPE-DOM groups of molecules and specific DOM fluorescence or absorption parameters. Furthermore, we have established, for the first time, empirical multiple regression models to estimate molecular parameters from optical measurements in open ocean waters.

## Electronic supplementary material

Supplementaty Information
