# Supplementary material for: Linking optical and molecular signatures of dissolved organic matter in the Mediterranean Sea
Source: Sci Rep. 2017 Jun 13;7:3436. doi: 10.1038/s41598-017-03735-4 (PMC5469803; doi:10.1038/s41598-017-03735-4)
Supplement: Supplementary file 1 — Supplementaty Information [file 41598_2017_3735_MOESM1_ESM.pdf]

## Supplementary Information

# Linking optical and molecular signatures of dissolved organic matter in the Mediterranean Sea

Alba María Martínez-Pérez[a,*], Mar Nieto-Cid[a], Helena Osterholz[b], Teresa S. Catalá[a,c], Isabel Reche[c], Thorsten Dittmar[b], Xosé Antón Álvarez-Salgado[a]

[a] Consejo Superior de Investigaciones Científicas - Instituto de Investigacións Mariñas (CSIC-IIM), Vigo, Spain

[b] Research Group for Marine Geochemistry, Institute for Chemistry and Biology of the Marine Environment (ICBM), Carl von Ossietzky University, Oldenburg, Germany

[c] Departamento de Ecología and Instituto del Agua, Universidad de Granada, Granada, Spain

## Contents of this file

Figures S1 to S6
Tables S1 to S2

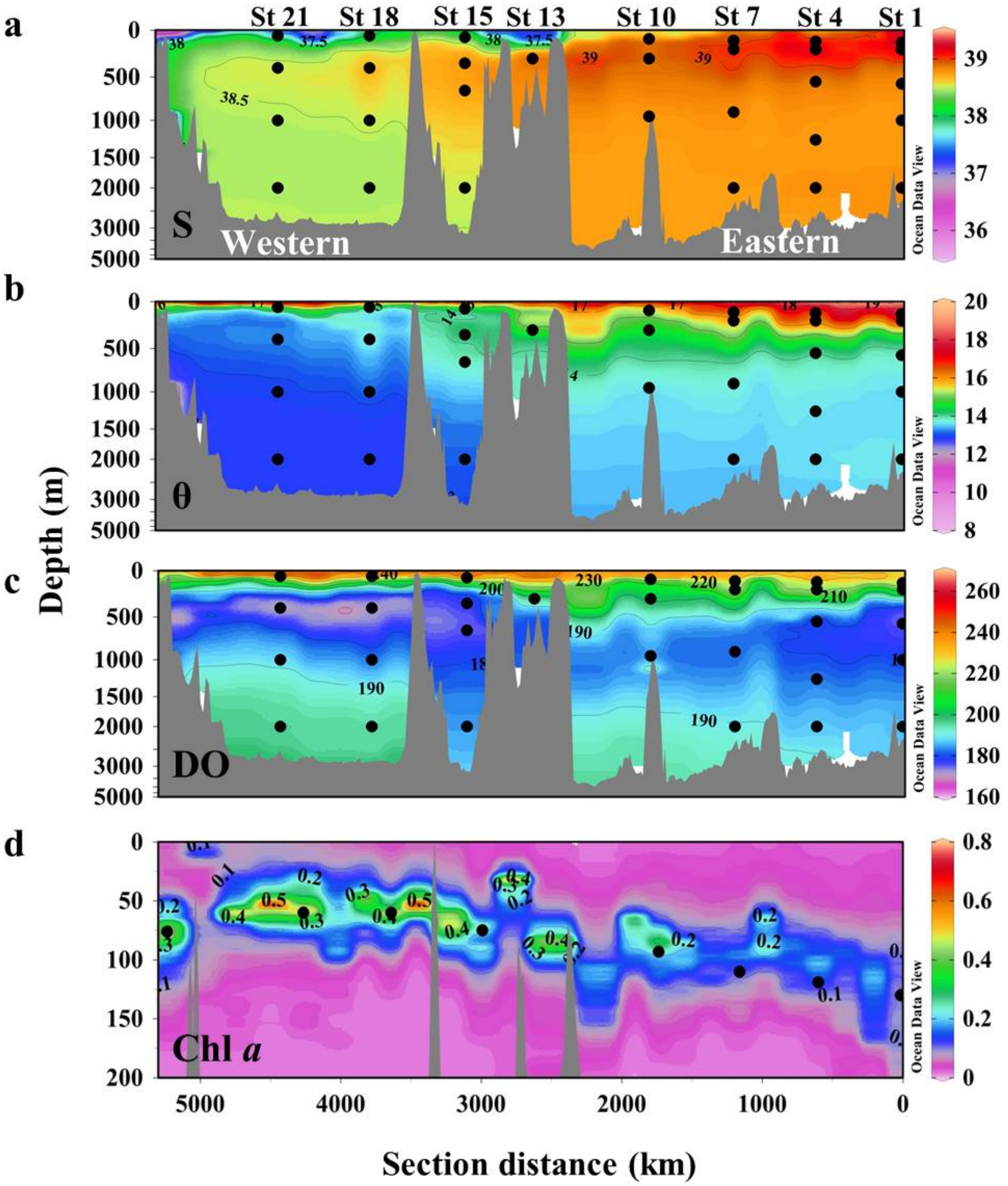


**Figure S1.** Distribution of (a) salinity (S), (b) potential temperature (θ) in ºC, (c) dissolved oxygen (DO) in µmol/Kg and (d) fluorescence of chlorophyll *a* (Chl *a*) in mg $m^{-3}$ obtained from the sensors attached to the rosette sampler along the Mediterranean Sea. Black circles represent the depths where samples were taken. Note that the depth is displayed on a not linear scale. Values from all stations were used to show these distributions. Figure created using Ocean Data View (R. Schlitzer, http://odv.awi.de).

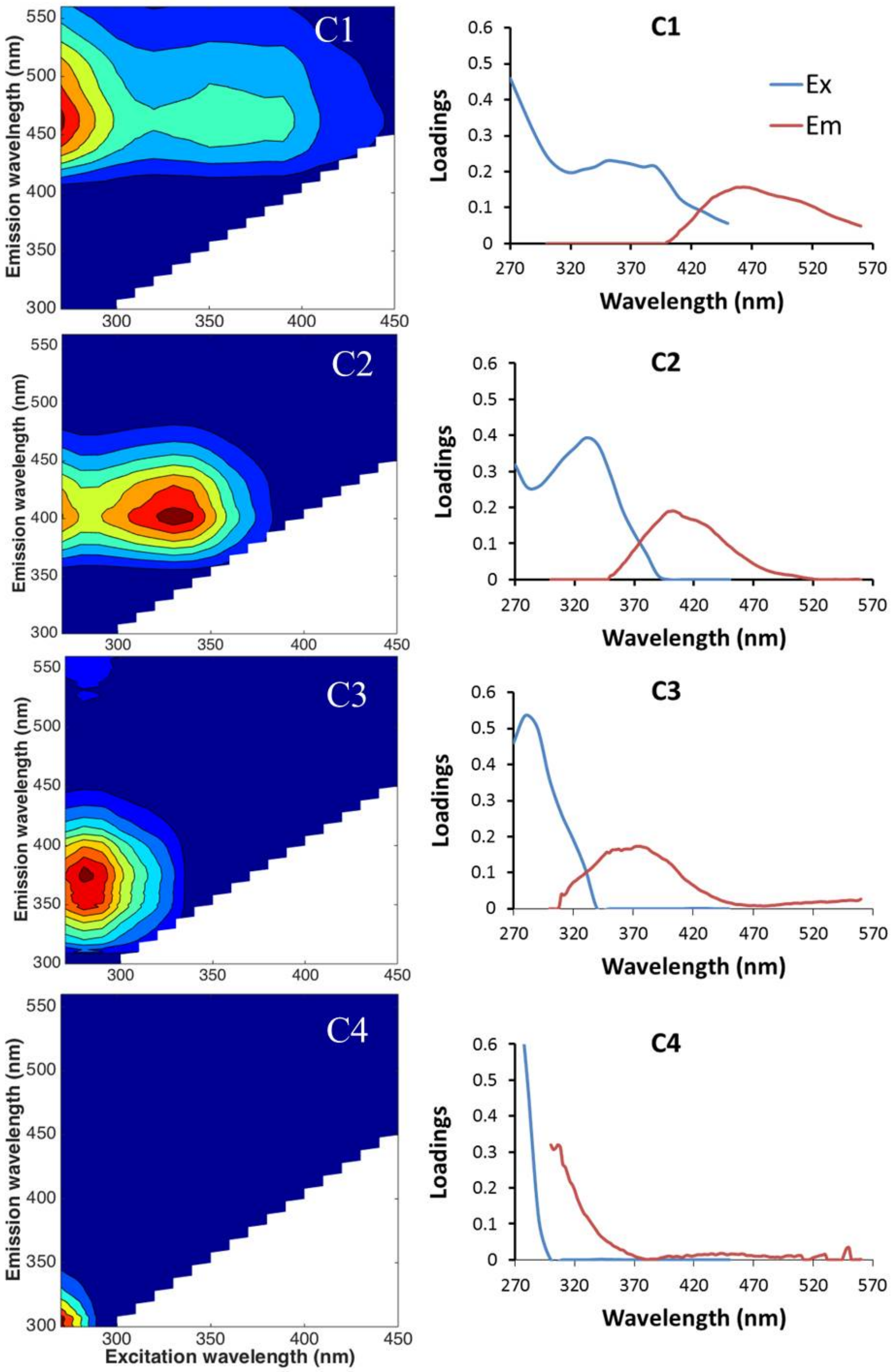


**Figure S2.** Fluorescence matrices of the four identified PARAFAC components. C1 represents a combination of classical defined peak A and C, C2 tracks classical defined peak M, C3 corresponds to peak T and C4 falls near peak B. The right panels represent the excitation (blue lines) and the emission (red lines) fluorescence intensities of the four PARAFAC components. Figure created using the software Matlab (version R2014B; MathWorks, USA).

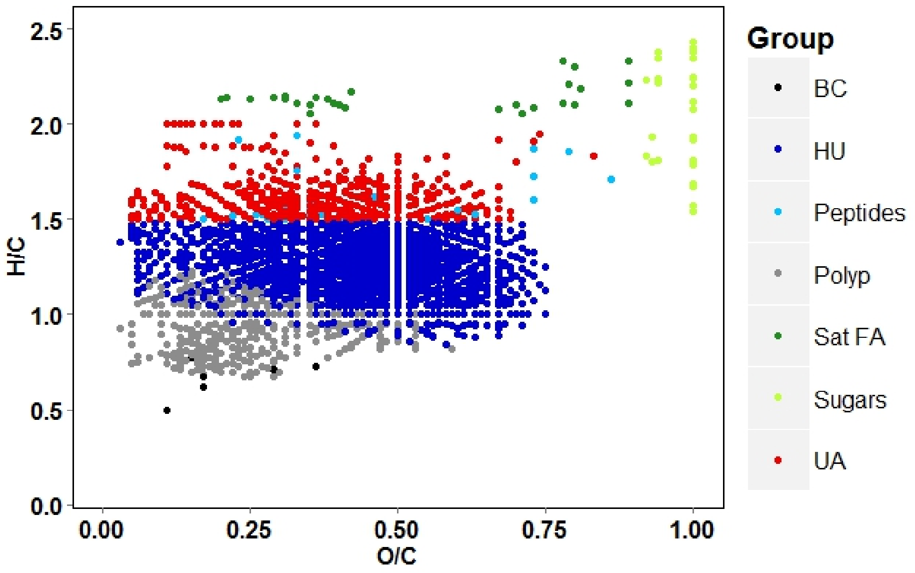


**Figure S3.** Van Krevelen diagram (O/C, oxygen to carbon and H/C, hydrogen to carbon ratios) for all formulae detected by the FT-ICR-MS sorted by compound groups**.** BC = Black carbon ($AI_{mod} > 0.66$), Polyp = polyphenols ($0.5 < AI_{mod} < 0.66$), HU = highly unsaturated compounds ($AI_{mod} < 0.5$, $H/C < 1.5$ and $O/C < 0.9$), UA = unsaturated aliphatic compounds ($1.5 \leq H/C < 2$, $O/C < 0.9$ and $N = 0$), peptides ($1.5 < H/C < 2$, $O/C \leq 0.9$ and $N > 0$), sugars ($O/C > 0.9$), Sat FA = saturated fatty acids ($H/C \geq 2$, $O/C < 0.9$). In addition, in this work we defined CRAM (carboxyl-rich alicyclic molecules; $0.3 < DBE/C < 0.68$, $0.2 < DBE/H < 0.95$ and $0.77 < DBE/O < 1.75$)[63]. Note that CRAM were not included in the van Krevelen plot because of the overlap with the highly unsaturated compounds. Figure created using the software R (version 3.1.1, 2014-07-10, [http://cran.r-project.org/])

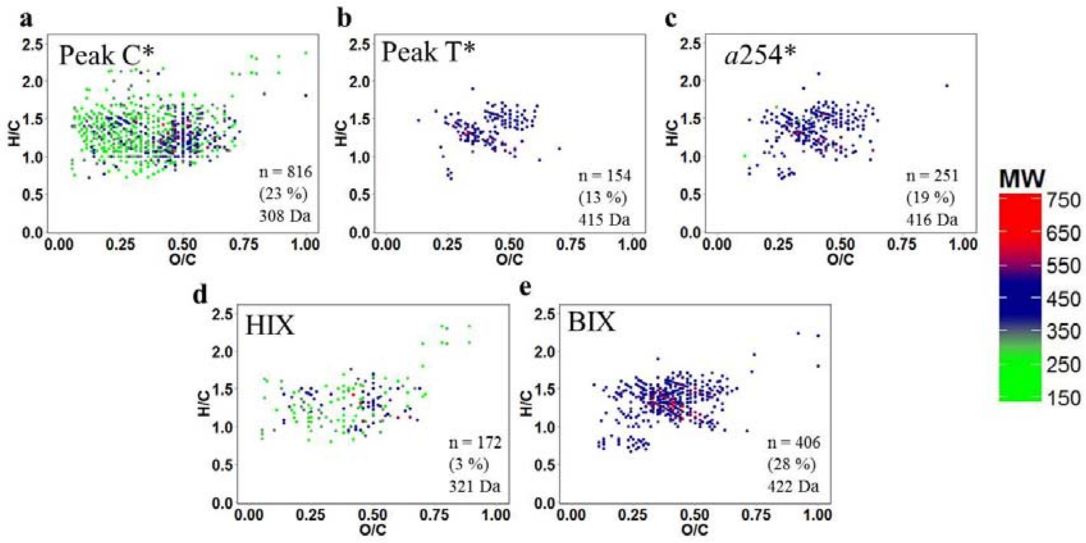


**Figure S4.** Van Krevelen diagrams showing the molecular formulae negatively correlated with: (a) peak C*, (b) peak T*, (c) a254*, (d) HIX and (e) BIX. Color scale represents the molecular weight. In the left corner of each panel is summarized the molecular formulae number correlating, the percentage of peak intensity and the intensity-weighted average molecular weight. Figure created using the software R (version 3.1.1, 2014-07-10, [http://cran.r-project.org/])

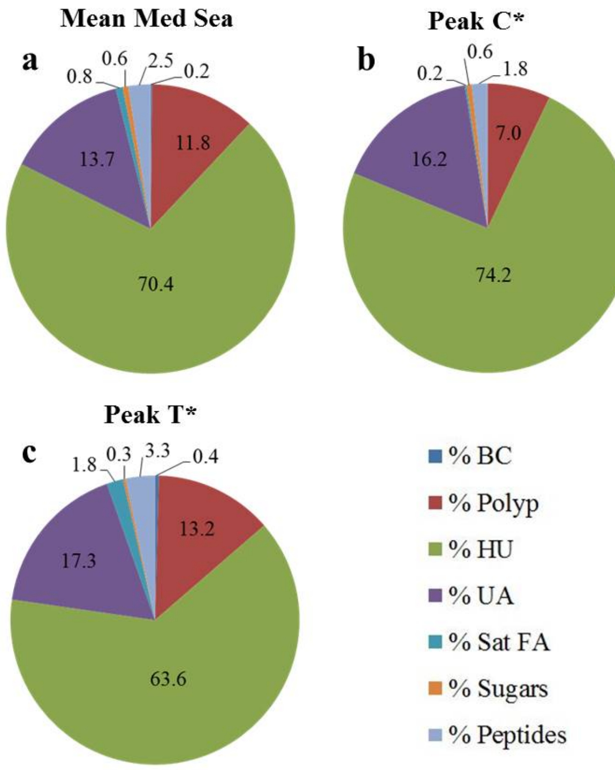


**Figure S5.** Pie charts showing the mean percentage distribution of the different groups of molecules (BC = Black carbon, Polyp = polyphenols, HU = highly unsaturated compounds, UA = unsaturated aliphatic compounds, peptides, sugars, Sat FA = saturated fatty acids) from the FT-ICR-MS analysis in (a) the Mediterranean Sea (Med Sea) and the distribution of the positively correlated molecular groups percentage according to the Spearman's rank correlations for (b) the carbon-specific fluorescence intensity of humic-like substances (peak C*) and (c) protein-like substances (peak T*). Figure created using Microsoft Excel 2010.

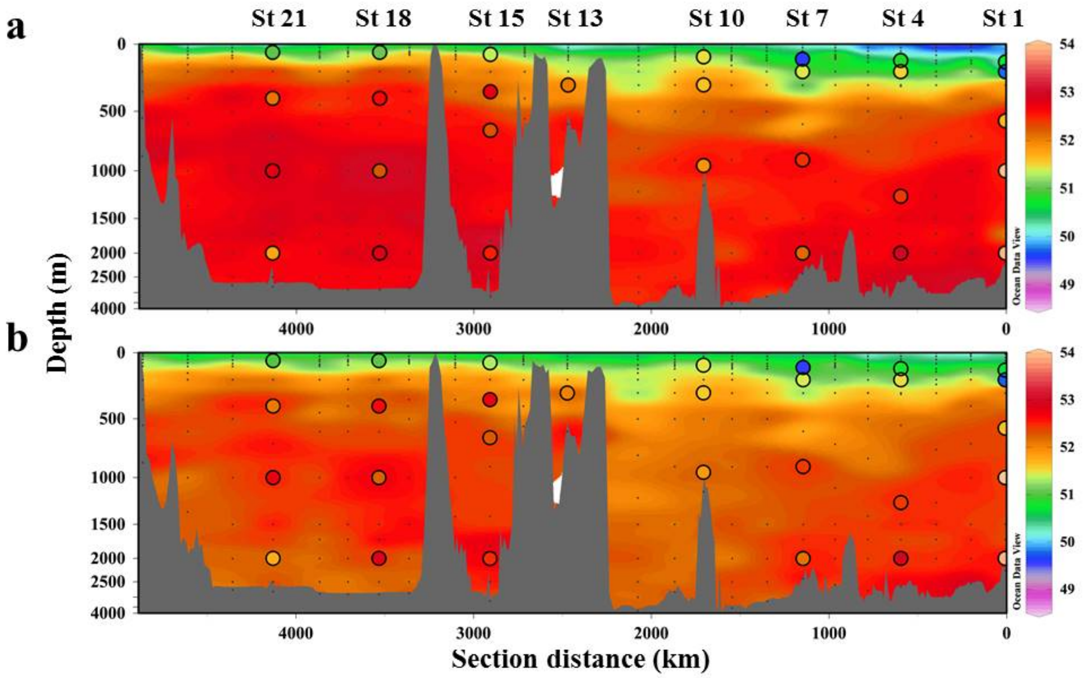


**Figure S6.** Estimations of the distributions of carboxyl-rich alicyclic molecules (CRAM) in % for the whole Mediterranean Sea determined from fluorescence measurements using (a).a model including all samples and (b) a model discarding the samples collected at St 1 (LIW, 1000 and 2000 m) and St 7 (110 m) which were more deviated from the linear regression line in the scatter plot of the modeled and measured values. Note that the patterns in both plots were similar. Figure created using Ocean Data View (R. Schlitzer, http://odv.awi.de).

**Table S1.** Negative correlations between the total assigned molecular formulae as well as the different type of molecular groups and the optical parameters; carbon-specific fluorescence intensity in NFIU L mg$^{-1}$ C of general humic-like substances (peak A*), terrestrial humic-like substances (peak C*), marine humic-like substances (peak M*), protein-like substances (peak T*), fluorescence ratios (peak A/T, C/T and M/T ratios), carbon-specific absorption coefficient at 254 nm (*a*254*; L m$^{-1}$ mg$^{-1}$ C), fluorescence index (FI) calculated as the ratio of emission at 470 and 520 nm at excitation wavelength 370 nm, freshness index (FrI) calculated as the ratio between 380 and the maximum intensity between 420 and 435 nm at 310 emission wavelength , biological index (BIX) determined as the ratio of emission at 380 and 430 nm at 310 nm of excitation wavelength and the humification index (HIX) calculated as the ratio between the integrated emission spectra between 435 and 480 and 300-345 nm at 260 nm of excitation wavelength. NC = no correlation. MW= intensity-weighted average molecular weight. Numbers in parentheses correspond to the signal intensity percentage from the spectra.

| | All | NC | Peak A* | Peak C* | Peak M* | Peak T* | A/T | C/T | M/T | C/M | *a*254* | FI | FrI | BIX | HIX |
|---|---|---|---|---|---|---|---|---|---|---|---|---|---|---|---|
| Nr total formulae | 3689 | 2071 | 772 | 816 | 801 | 154 | 877 | 872 | 880 | 65 | 251 | 388 | 453 | 406 | 172 |
| | (100) | (37) | (21) | (23) | (23) | (13) | (24) | (24) | (24) | (1) | (19) | (26) | (31) | (28) | (4) |
| MW (Da) | 375 | 373 | 309 | 309 | 309 | 416 | 314 | 315 | 315 | 422 | 417 | 421 | 419 | 422 | 321 |
| Nr formulae with N | 1457 | 673 | 363 | 384 | 378 | 50 | 411 | 408 | 410 | 19 | 80 | 134 | 159 | 140 | 71 |
| | (100) | (45) | (27) | (28) | (28) | (6) | (29) | (29) | (29) | (1) | (8) | (11) | (13) | (11) | (4) |
| Black Carbon (Nr) | 8 | 3 | 3 | 3 | 3 | 0 | 4 | 4 | 4 | 1 | 0 | 0 | 0 | 0 | 0 |
| | (100) | (23) | (50) | (50) | (50) | (0) | (62) | (62) | (62) | (13) | (0) | (0) | (0) | (0) | (0) |
| Polyphenols (Nr) | 433 | 195 | 119 | 125 | 122 | 5 | 134 | 135 | 138 | 3 | 17 | 29 | 41 | 34 | 27 |
| | (100) | (41) | (19) | (23) | (20) | (2) | (22) | (23) | (24) | (0) | (6) | (14) | (19) | (15) | (3) |
| Highly unsaturated (Nr) | 2594 | 1288 | 474 | 499 | 490 | 110 | 544 | 537 | 542 | 57 | 163 | 292 | 318 | 281 | 102 |
| | (100) | (30) | (19) | (20) | (20) | (14) | (22) | (21) | (22) | (1) | (20) | (29) | (33) | (29) | (4) |
| Unsaturated aliphatic (Nr) | 507 | 210 | 134 | 141 | 142 | 39 | 150 | 151 | 150 | 4 | 67 | 50 | 81 | 80 | 28 |
| | (100) | (25) | (26) | (29) | (29) | (18) | (28) | (28) | (28) | (0) | (25) | (20) | (31) | (30) | (6) |
| Saturated fatty acids (Nr) | 28 | 9 | 17 | 17 | 17 | 0 | 17 | 17 | 17 | 0 | 1 | 1 | 0 | 0 | 7 |
| | (100) | (8) | (88) | (88) | (88) | (0) | (88) | (88) | (88) | (0) | (3) | (1) | (0) | (0) | (21) |
| Sugars (Nr) | 30 | 18 | 2 | 2 | 2 | 0 | 2 | 2 | 2 | 0 | 1 | 7 | 4 | 3 | 0 |
| | (100) | (28) | (8) | (8) | (8) | (0) | (8) | (8) | (8) | (0) | (3) | (56) | (40) | (20) | (0) |
| Peptides (Nr) | 89 | 40 | 23 | 25 | 25 | 0 | 26 | 26 | 27 | 0 | 2 | 9 | 9 | 8 | 8 |
| | (100) | (32) | (41) | (41) | (41) | (0) | (42) | (42) | (43) | (0) | (2) | (17) | (17) | (16) | (19) |

**Table S2.** Total number of molecular formulae with shared correlations (positive and negative) between each optical variable.

| | Peak A* | Peak C* | Peak M* | Peak T* | a254* | BIX | HIX |
|---|---|---|---|---|---|---|---|
| Peak A* | 1180 | | | | | | |
| Peak C* | 1159 | 1327 | | | | | |
| Peak M* | 1140 | 1233 | 1233 | | | | |
| Peak T* | 743 | 743 | 734 | 858 | | | |
| a254* | 817 | 817 | 808 | 796 | 969 | | |
| BIX | 1053 | 1068 | 1041 | 819 | 922 | 1249 | |
| HIX | 319 | 312 | 299 | 272 | 302 | 357 | 470 |
